# Editors’ Book Table

**Published:** 1873-05

**Authors:** 


### Editors' Book Table.

[NOTE.—All works reviewed in the columns of the CHICAGO MEDICAL JOURNAL may be found in the extensive stock of W. B. KEEN, COOKE & CO., whose catalogue of Medical Books will be sent to any address upon request.]

BOOKS RECEIVED.

*Text Book of Physiology, General, Special and Practical.* By JOHN HUGHES BENNETT, M.D., F.R.S.E., Professor of the Institutes of Medicine or Physiology, and senior Professor of Clinical Medicine in the University of Edinburgh, etc. With twenty-one Photo-Lithographic Plates. Pp. 606, with twenty-one pages of Illustrations, and twenty-four of Descriptive Text. 12mo. Extra cloth, $3.50.

Any book from this veteran teacher will be sure to attract attention and secure a sale, and the one before us, containing, as it does, the results of the long continued and careful investigations of a skilled observer and experimenter, cannot fail of wide popularity. Professor Bennett has been a teacher of systematic Physiology since 1848, and his particular views are very generally understood by students of Physiology. We find the subject divided in discussion into three parts, viz:

GENERAL PHYSIOLOGY, including—1. *Chemistry of the Tissues.* 2. *General Histology.* 3. *Physical and Vital Properties of the Tissues;* with a chapter on *Life or Vitality.*

SPECIAL PHYSIOLOGY, including—1. *Nutrition.* 2. *Innervation.* 3. *Reproduction;* with a section on *Death.*

PRACTICAL PHYSIOLOGY, discussing—1. *Practical Chemical Physiology.* 2. *Practical Histological Physiology.* 3. *Practical Experimental Physiology.*

The illustrations are photo-lithographic, and the book, as a whole, presents the greatest amount of information it has been our fortune to find condensed in the same bulk. We most heartily commend it both as a text book for students and for reference by practitioners.

We notice that Professor Bennett gives prominence in his account of the blood corpuscles to the important discovery of Professor Freer (CHICAGO MEDICAL JOURNAL, *April* 15, 1869,) with regard to the shape of these bodies, and also shows the appearance in his plates.

*Diseases of the Urinary Organs;* including Stricture of the Urethra, Affections of the Prostate, and Stone in the Bladder. By JOHN W. S. GOULEY, M.D., Surgeon to Bellevue Hospital, etc. With 103 Wood Engravings. New York: William Wood & Co. 1873. Cloth, 8vo. Pp. 368.

Dr. Gouley herein gives the substance of his Lectures at Bellevue Hospital, and in the Medical Department of the University of New York. It is intended to embody essentially the results of his experience in both his public and private practice, but also gives merited reference to the teachings of many others eminent in this department of surgical practice. It is a manual of the pathogeny, clinical history and treatment of some of the graver diseases of the urinary organs, with suggestions as to their early detection.

Writing largely from the standpoint of his own observation, the author recounts his cases with great clearness and accuracy, and displays, not only familiarity with the topics reviewed, but much ingenuity and originality in the adaptation of the instruments and mechanical appliances necessary for the successful management of the various difficulties encountered. It is a valuable contribution to the literature of the subject, and the most skilled surgeon will certainly find in it many important ideas for subsequent practice.

*Expression: Its Anatomy and Philosophy.* By SIR CHARLES BELL, K.H. With the Original Notes and Illustrations designed by the Author; and with additional Illustrations and Notes by the Editor of the "Phrenological Journal." An entirely new and enlarged Edition. New York: Samuel R. Wells, Publisher, No. 389 Broadway. 1873. Cloth. 12mo. Pp. 201. $1.50.

*Clinical Lectures on various Important Diseases.* Being a collection of the Clinical Lectures delivered in the Medical Wards of Mercy Hospital, Chicago. By NATHAN S. DAVIS, A.M., M.D., Professor of Principles and Practice of Medicine and Clinical Medicine, in Chicago Medical College. Edited by Frank H. Davis, M.D. Chicago: J. J. Spalding & Co., 138 Clark Street. 1873. Cloth. 12mo. Pp. 265. $3.00.

Eighteen lectures, covering quite a variety of important topics, are here collected. We find two on Continued Fever, two on the Summer Complaints of Children, and one on each of the following: Periodical Fever; Rheumatic Fever; Scarlatina and Rubeola; Respiratory Affections; Pulmonary Tuberculosis; Diseases of the Alimentary Tract; Intestinal and Uterine Irritation; Dropsy; Causes of Cardiac Disease; Neuralgia; Nervous Affections; Affections of the Brain; Cerebro-Spinal Disease; Cutaneous Diseases.

The Clinical Reports containing these lectures have appeared from time to time in the columns of the *Medical Examiner*, but from the interest in them manifested by readers, it was deemed proper to collect them in a more convenient form. Although reported by different persons, little change has been made, save in occasional consolidation of two or three Clinics in one.

As containing the opinions and modes of practice of a gentleman well known to the profession, of long continued and remarkably extensive practice, this unpretentious volume is well worth the perusal of the medical public.

*Civil Malpractice.* A Report Presented to the Military Tract Medical Society, at the Fifteenth Semi-Annual Meeting, January, 1873. By M. A. MCCLELLAND, M.D. Chicago: W. B. Keen, Cooke & Co., Publishers, Nos. 113 & 115 State Street. 1873. Cloth. 8vo. $2.00.

Readers of the JOURNAL will recognize this as a collection in book form of the series of articles published in the present volume. So much interest has been exhibited in them that this was considered due the profession. Further notice will be given in a subsequent number.

*Archives of Ophthalmology and Otology.* Edited and Published simultaneously in English and German. By Prof. H. KNAPP, M.D., of New York, and Prof. S. MOOS, M.D., of Heidelberg. Volume III., No. 1. New York: William Wood & Co., 27 Great Jones Street. 1873. Paper. Pp. 260 and 5 pp. plates. $7.00 per year. Issued semi-annually. Price for the bound volumes already published, in half morocco, $8.50 each.

*New Life in New Lands.* Notes of Travel across the American Continent, from Chicago to the Pacific and back. By GRACE GREENWOOD. 12mo. Pp. 415. Cloth, Stamped Cover. Price, $2.00.

This is a gathered series of letters—racy, brilliant, piquant, full of keen observation, and pungent statement of facts, picturesque in delineation of scenes on the plains, in the mountains, and along the sea. The late William H. Seward characterized her account of Mormons and Mormonism, which has been included in the book, *the most graphic and trustworthy he had ever read.* It is a book full of delightful reading all the way through, and, like a nut, has many a choice morsel tucked away in sly corners for those who find them.

PAMPHLETS RECEIVED.

*Thirteenth Biennial Report of the Trustees, Superintendent and Treasurer of the Illinois State Hospital for the Insane at Jacksonville.* December, A. D. 1873.

*American Association for the Cure of Inebriates:* Proceedings of the Third Meeting, held in New York, October 8, 9 and 10, 1872.

*The Charter and By-Laws of the New York State Inebriate Asylum,* (Binghamton,) etc., etc. 1873.

*Report of Commissioners Appointed to Select a Site and Build an Asylum for the Insane of this State,* [New Jersey.] November 1st, 1872. With Ground Plan, etc.

*The Logic of Medicine.* By EDWARD S. DUNSTER, M.D. New York: D. Appleton & Co. 1873. An Address before the New York Academy of Medicine, December 30th, 1872, being its 29th Anniversary.

*Transactions of the Third Annual Session of the Medical Society of Virginia.* Staunton, November, 1872.

*Manufacturing, Agricultural and Industrial Resources of Iowa.* By H. S. HYATT, Editor of the "Iowa Progress."

*The Criminal Use of Proprietary Nostrums.* By ELY VAN DE WASKER, M.D., Syracuse, New York.

*Report of the Board of Administrators of the Charity Hospital to the General Assembly of Louisiana.* Session of 1873. From J. D. Lichtenberger.

*Report of the Pennsylvania Hospital for the Insane, for the year* 1872. By THOMAS S. KIRKBRIDE, M.D., Physician-in-Chief and Superintendent. Pp. 70.

*Florida and South Carolina as Health Resorts.* By WILLIAM W. MORLAND, M.D., Harv. Pp. 20.

*Contributions to the Diagnosis of Cancerous Diathesis—Carcinosis.* By WILLIAM B. NEFTEL, M.D., New York. A reprint.

*New Method of Preserving Tumors and certain Urinary Deposits During Transportation.* By JOSEPH G. RICHARDSON, M.D., etc., etc. Philadelphia: J. B. Lippincott & Co.

*A New Method of Treating Strictures of the Urethra, after External Sections.* By C. H. MASTIN, M.D., Mobile, Ala. Read by request before the Mobile Pathological Society, Sept. 9, 1872. From the author.

*Ninetieth Annual Catalogue of the Medical School* (Boston) *of Harvard University.* 1872–73.

*Transactions of the Territorial Medical Society at its Second Annual Meeting.* Held in Denver, Colorado, September, 1872.

*Proceedings of the Third Annual Session of the State Medical Association of Arkansas.* Held at Little Rock, Jan. 6, 7 and 8, 1873.

*Eighth Annual Report of the Illinois Institution for the Education of Feeble Minded Children, located at Jacksonville.* To the Governor of Illinois, Dec., 1872. Organized 1865—Incorporated 1871. From C. T. WILBUR, M.D., Superintendent.

